# Early outcomes of aortic valve replacement with Perceval PLUS sutureless valve: results of the prospective multicentric MANTRA study

**DOI:** 10.1186/s13019-024-02861-1

**Published:** 2024-06-21

**Authors:** Slobodan Micovic, Angelo Nobre, Jae Woong Choi, Marco Solinas, Sharaf-Eldin Shehada, Michele Torella, Cristian Baeza, Eugene Parrino, Francesco Pollari, Giovanni Troise, Utz Kappert, Friedrich Mellert, Hyung Gon Je, Vincenzo Argano, Ka Yan Lam, Mauro Rinaldi, Herbert Gutermann, Bart Meuris, the MANTRA Investigators

**Affiliations:** 1 Dedinje Cardiovascular Institute, Milana Tepica 1, Belgrade, 11000 Serbia; 2 Hospital de Santa Maria Lisbon, Lisbon, Portugal; 3 Seoul National University Hospital, Seoul, South Korea; 4 Ospedale del Cuore Di Massa, Massa, Italy; 5 University Hospital Essen, Essen, Germany; 6 University of Campania “L. Vanvitelli” - Monaldi Hospital, Naples, Italy; 7 University Hospitals Cleveland Medical Center, Cleveland, USA; 8 Ochsner Clinic Foundation, New Orleans, USA; 9 Klinikum Nürnberg-Paracelsus Medical University, Nuremberg, Germany; 10 Fondazione Poliambulanza Istituto Ospedaliero, Brescia, Italy; 11 Herzzentrum Dresden GmbH Universitätsklinik, Dresden, Germany; 12 Klinikum Oldenburg GGMBH AoR, Oldenburg, Germany; 13 Pusan National University Yangsan Hospital, Yangsan, South Korea; 14 Policlinico Paolo Giaccone, Palermo, Italy; 15 Catharina Ziekenhuis, Eindhoven, The Netherlands; 16 A.O.U. Città Della Salute E Della Scienza Di Torino - Ospedale Molinette, Turin, Italy; 17 Ziekenhuis Oost Limburg, Genk, Belgium; 18 UZ Leuven, Louvain, Belgium

**Keywords:** Aortic valve replacement, Sutureless valve, Real-life data, Perceval plus

## Abstract

**Background:**

The aim of this study is to report the preliminary real-word clinical and hemodynamic performance from the MANTRA study in patients undergoing aortic valve replacement with Perceval PLUS sutureless valve.

**Methods:**

MANTRA is an ongoing “umbrella” prospective, multi-center, international post-market study to collect real-life safety and performance data on Corcym devices (Corcym S.r.l, Saluggia, Italy). Clinical and echocardiographic outcomes were collected preoperatively, at discharge and at each follow up. KCCQ-12 and EQ-5D-5L quality of life questionnaires were collected preoperatively and at 30-days.

**Results:**

A total of 328 patients underwent aortic valve replacement with Perceval PLUS in 29 International institutions. Patients were enrolled from July 2021 to October 2023 and enrollment is still ongoing. Mean age was 71.9 ± 6.4 years, mean EuroSCORE II was 2.9 ± 3.9. Minimally invasive approach was performed in 44.2% (145/328) of patients; concomitant procedures were done in 40.8% (134/328) of cases. Thirty-day mortality was 1.8% (6**/**328) and no re-interventions were reported. Pacemaker implant was required in 4.0% (13/328) of the patients.

The assessment of the functional status demonstrated marked and stable improvement in NYHA class in most patients at 30-day follow-up, with significant increase of KCCQ-12 summary score (from 58.8 ± 23.0 to 71.8 ± 22.1, *p* < 0.0001) and EQ-5D-5L VAS score (from 64.5 ± 20.4 to 72.6 ± 17.5, *p* < 0.0001). Mean pressure gradient decreased from 46.2 ± 17.3 mmHg to 10.1 ± 4.7 mmHg at 30-day follow-up. Low or no incidence of moderate-to-severe paravalvular or central leak was reported.

**Conclusions:**

Preliminary results demonstrate good clinical outcomes and significant improvement of Quality of Life at 30-days, excellent early hemodynamic performance within patient implanted with Perceval PLUS.

**Trial Registration:**

The MANTRA study has been registered in ClinicalTrials.gov (NCT05002543, Initial release 26 July 2021).

**Supplementary Information:**

The online version contains supplementary material available at 10.1186/s13019-024-02861-1.

## Background

Aortic valve stenosis is the most frequent heart valve disease requiring surgical intervention in the western community [1]. Despite recent developments and promising clinical results in catheter-based valve implantation, [2, 3] open-heart surgery with aortic valve replacement (AVR) remain the gold standard treatment for patients presenting with severe aortic valve disease in low to intermediate risk patients [1, 4]. According to the Society of Thoracic Surgeons (STS) database, the operative risk of AVR has dramatically improved in the last decade, showing a reduction of mortality from 4.3% to 2.6% [5, 6]. Despite these results, elderly patients and patients with significant comorbidities referred for AVR are still at higher risk for conventional surgery.

Traditionally, the surgical options for AVR have been confined to the choice between mechanical and biological prostheses [1]; however, biological prosthetic valves have undergone major advances in valve design and implantation techniques, largely in relation to transcatheter and minimally invasive access [7]. In this scenario of rapid development of new valve technologies, sutureless aortic valve has gained interest. The Perceval valve (Corcym S.r.l., Saluggia, Italy) is a self-expandable, sutureless, surgical aortic bioprosthesis and with promising results in terms of mortality, morbidity and hemodynamic performances [8–11]. The Perceval PLUS model features an innovative tissue treatment (FREE), that addresses both sources of tissue mineralization (phospholipids and aldehydes) [12].

The aim of this study is to report preliminary real-word clinical and hemodynamic performance from the MANTRA study (CORCYM Mitral, Aortic aNd Tricuspid Post-maRket Study in a reAl-world Setting) in patients who underwent AVR using the Perceval Plus valve.

## Patients and methods

### Study design

Details about the design of the MANTRA Study have been published [13]. The MANTRA study is a prospective, global, post-marketing clinical follow-up study. The aim is to collect safety and device performance data covering the Corcym cardiac surgery portfolio for the treatment of aortic, mitral, and tricuspid valve diseases. The study uses a master protocol that outlines the main common parameters and specific questions are addressed in three substudies: aortic, mitral/tricuspid and Memo 4D. The study concept development and data collection have been extensively supported by a Steering Committee consisting of expert cardiac surgeons and cardiologists.

The study allows for inclusion of isolated as well as combined procedures with multiple device implantation (Sponsor or non-Sponsor devices), with the possibility to collect safety and performance information on all the Corcym devices implanted as well as to collect data on the interaction with concomitant valve treatments.

All endpoints are defined according to the most recent guidelines for heart valve procedures [14, 15]. Hemodynamic and structural performance from echocardiographic findings is being collected preoperatively, intra-operatively, at discharge, at 30-days (+ 14 days), at 12 months after implant and at each subsequent follow-up. Data are collected as per the recommendations for the imaging assessment of prosthetic heart valves [16]. Additionally, procedural and hospitalization information are collected, including Enhanced Recovery after Surgery (ERAS) in sites using such protocols, and patient outcome measures such as New York Heart Association (NYHA) classification and quality-of-life questionnaires.

This study was approved by the Ethics Committees/Institutional Review Board and/or health authorities according to local regulations. No more than 30 patients are planned to be enrolled at any given site, as per Investigational Protocol.

### Study procedures

This interim report focuses on subjects included in the aortic sub-study, diagnosed with aortic valve disease who were considered suitable to undergo AVR with Perceval PLUS. All subjects were required to provide informed consent before undergoing any clinical investigation-specific assessments and treatments.

The schedule follows common standard-of-care practices and may vary across sites.

Patients are evaluated preoperatively, during operative procedure, at hospital discharge and at 30-days. Annual evaluations are planned until the 10-year follow-up and will be completed for all available patients. All patients undergo a clinical evaluation and participants are asked to complete quality-of-life questionnaires (Kansas City Cardiomyopathy Questionnaire-12 [KCCQ-12] [17] and EQ-5D-5L [18]) at baseline, 30-day and 1-year follow-up. Hemodynamic and structural performance is based on site-reported echocardiographic evaluations.

The study data are collected using a web-based electronic data capture system (Merative, Ann Arbor, Michigan, USA). The data are continuously reviewed for omissions, errors and values requiring further clarification, via computerized and manual procedures. Serious adverse events, medical history and concomitant medications are coded using standard dictionaries and reported once diagnosed. More patients are under enrollment.

### Study device

**Fig. 1:**
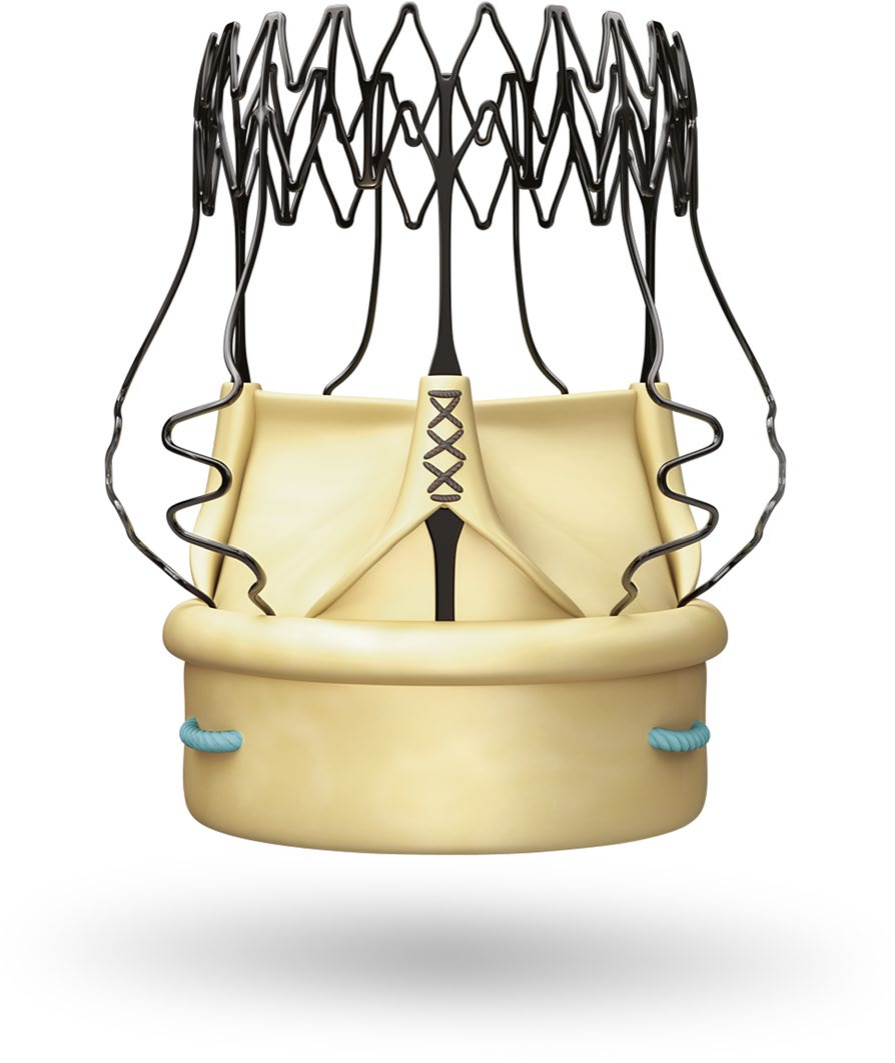
Perceval Plus valve

The Perceval PLUS valve (Fig. 1), based on the Perceval platform, is a self-anchoring, self-expanding, sutureless, surgical aortic bioprosthesis indicated for the replacement of malfunctioning or degenerated native aortic heart valves or prostheses. This bioprosthesis has a functional component, comprising bovine pericardium, stabilized in a buffered glutaraldehyde solution and a super-elastic Nitinol stent, which has the dual role of supporting the valve and anchoring it to the aortic root with no permanent sutures. The valve is stored in an aldehyde-free solution, and no rinse is required before implantation. The valve is available in four sizes: S, covering annuli of 19–21 mm, M for annuli 21–23 mm, L for annuli 23-25 mm, and XL covering annuli of 25-27 mm. With respect to the Perceval platform, the Perceval Plus valve features an innovative tissue treatment (FREE) that addresses both sources of tissue mineralization (phospholipids and aldehydes) [12]. Additionally, the length on the connecting struts between the two waves of the inflow ring in the size XL was shortened of 1 mm to reduce of the protrusion of the valve below the aortic annulus with the aim to decrease the risk of impairment of the atrio-ventricular conduction system. The valve sizing procedure is performed with dedicated sizers that features two obturators: one white and one transparent. Compared to the initial experiences in which the surgeons were recommended by the manufacturer to choose a larger valve size when the white obturator was passing with resistance, the current sizing recommendation advises that the transparent obturator should pass without resistance and the white one should pass with a light friction. The valve is implanted through dedicated new generation accessories (RelyON system), with improved ease of use and optimized visibility during implant. Prior to implantation, the prosthesis diameter is collapsed to a suitable size for loading it onto a delivery system. The valve is then positioned and released in the aortic root under direct visualization and subsequently post-dilated using a dedicated balloon catheter.

### Statistical analysis

Variables are described as mean ± standard deviation or median and inter quartile ranges (quartile ranges 25th and 75th) for continuous variables and as number and percent (%) for categorical variables. Outcomes are reported as descriptive statistics. The proportions of early adverse events were calculated as the total number of events divided by the total number of patients. Changes in KCCQ and EQ-5D-5L scores from baseline were evaluated at 30-days using unpaired t-tests. The statistical analyses were performed using SAS® (Release 9.4, by SAS Institute Inc., Cary, NC, USA).

## Results

### Patient and operative data

Between July 2021 and October 2023, 328 patients underwent AVR with the Perceval PLUS valve in 29 institutions. As detailed in Table 1, 166 patients (50.6%) were female. The overall mean age was 71.9 ± 6.4, with 14.3% (47) of the patients below 65 years. The mean EuroSCORE II was 2.9 ± 3.9. Most patients (283/324, 87.3%) were in NYHA class II or III. The etiology of aortic valve disease was degenerative in most of the cases (293, 89.3%), with 97.9% (321) of patients presenting stenosis or steno-insufficiency. In 14.9% (49) of the cases, the native valve was bicuspid, predominantly Sievers Type 1 (37, 75.5%).

**Table 1:** Baseline characteristics

Parameters	Overall population (*N* = 328)
**Age [years] (mean ± SD)**	71.9 ± 6.4
**Age by class, *N* (%)**	
**< 65 years**	47 (14.3%)
**≥ 65 years**	281 (85.7%)
**Female, N (%)**	166 (50.6%)
**BSA [m**^**2**^**] (mean ± SD)**	1.9 ± 0.2
**EuroSCORE II [%] (mean ± SD)**	2.9 ± 3.9
**STS score [%] (mean ± SD)**	2.6 ± 2.2
**NYHA class II–III, *N* (%)**	283/324 (87.3%)
**Comorbidities, N (%)**	
Arrhythmia	60 (18.3%)
Heart failure	39 (11.9%)
Previous CVA	23 (7.0%)
Dyslipidemia	218 (66.5%)
CAD	141 (43.0%)
Hypertension	245 (74.7%)
Obesity	90 (27.4%)
Diabetes	111 (33.8%)
Left ventricular hypertrophy	106 (32.3%)
Myocardial infarction	29 (8.8%)
Peripheral vascular disease	36 (11.0%)
Pulmonary hypertension	35 (10.7%)
Renal failure	37 (11.3%)
Tobacco user	111 (33.8%)
**Previous surgery, *N* (%)**	
Aortic Valve Repair	1 (0.3%)
Aortic Root Replacement	3 (0.9%)
Aortic Valve Replacement	12 (3.7%)
AF treatment	10 (3.0%)
Congenital Defect Repair	1 (0.3%)
CABG	5 (1.5%)
Mitral Valve Repair	3 (0.9%)
Mitral Valve Replacement	4 (1.2%)
Mitral Valve Ring	2 (0.6%)
Pacemaker/defibrillator implantation	12 (3.7%)
PCI	39 (11.9%)
Tricuspid Valve Repair	1 (0.3%)
**Bicuspid valve, *N* (%)**	49 (14.9%)
Sievers Type 0	4 (8.2%)
Sievers Type 1	37 (75.5%)
Sievers Type 2	5 (10.2%)
Not reported	3 (6.1%)
**Etiology, *N* (%)**	
Congenital	10 (3.0%)
Degenerative	293 (89.3%)
Endocarditis	2 (0.6%)
Rheumatic disease	10 (3.0%)
Other^a^	13 (4.0%)
Stenosis, N (%)	155 (47.3%)
Insufficiency, N (%)	7 (2.1%)
Steno-Insufficiency, N (%)	166 (50.6%)

^a^Redo, Degenerative + Congenital, Pannus

*CABG* Coronary Artery Bypass Graft, *CVA* Cardiovascular accident, *CAD* Coronary Artery Disease,  *AF* Atrial Fibrillation, *PCI* Percutaneous Coronary Intervention

Operative data are reported in Table 2. Almost half of the patients (145, 44.2%) underwent AVR through minimally invasive approach (141, 72.7% in isolated AVR), mini-sternotomy in 72 cases (49.7%) and mini-thoracotomy in 73 cases (50.3%). Concomitant procedures were performed in 134 patients (40.8%); in most cases (94, 28.7%), a coronary artery bypass graft (CABG) was performed, while a mitral or tricuspid valve procedure was done in 2.7% (9) and 1.8% (6) of patients, respectively.

**Table 2:** Operative data

Parameters	Overall population (*N* = 328)
**Surgical approach, N (%)**	
Median sternotomy	183 (55.8%)
Minimally Invasive	145 (44.2%)
Mini sternotomy	72 (49.7%)
Mini thoracotomy	73 (50.3%)
**Minimally invasive approach in isolated AVR, *N* (%)**	141/194 (72.7%)
**Concomitant Procedures**^**a**^**, *N* (%)**	
Mitral Valve Repair/replacement	9 (2.7%)
**Repair**	**2 (0.6%)**
**Replacement**	**7 (2.1%)**
Tricuspid Valve Repair/replacement	6 (1.8%)
CABG	94 (28.7%)
AF Treatment	31 (9.5%)
Septal Myectomy	2 (0.6%)
Atrial Septal Defect Repair	2 (0.6%)
Aortic Root Replacement	5 (1.5%)
Other^b^	26 (7.9%)
**CPB time [min] (mean ± SD) _Overall**	92.9 ± 44.2
**Cross-clamp time[min] (mean ± SD)_Overall**	61.0 ± 28.8
**CPB time [min] (mean ± SD) _Isolated AVR_MICS approach**	82.5 ± 30.5
**Cross-clamp time[min] (mean ± SD)_Isolated AVR_MICS approach**	52.8 ± 17.7
**Perceval PLUS sizes, *N* (%)**	
Small	65 (19.8%)
Medium	98 (29.9%)
Large	98 (29.9%)
Extra large	67 (20.4%)
**Total length of stay (days), median (IQR)**	10 (8–14)
**ICU stay (days), median (IQR)**	2 (1–4)
**Ventilation time (hours), median (IQR)**	9 (6–12)

^a^one subject can have more than one concomitant procedure; ^b^LAA closure/ligation, Ascending Aorta replacement, Tricuspid and Mitral Valve repair without ring

*AVR* Aortic Valve Replacement, *TVr* Tricuspid Valve Repair, *CABG* coronary artery bypass graft, *IQR* Interquartile range

The Perceval PLUS valve was successfully implanted in all patients even though in 9 cases the first attempt failed due to initial mispositioning or mis-sizing of the bioprosthesis or the native annulus, accordingly. The implanted valve size was Small (S) in 19.8% (65) of patients, Medium (M) and Large (L) in 29.9% (98), and Extra-large (XL) in 20.4% (67) of patients. The overall mean cardiopulmonary bypass (CPB) time was 92.9 ± 44.2 min and the mean aortic cross-clamp time was 61.0 ± 28.8 min; considering the cases of isolated AVR in minimally invasive approach (141, 72.7%), the CPB time and the mean aortic cross-clamp time were 82.5 ± 30.5 min and 52.8 ± 17.7 min, respectively. The median length of hospital stay was 10 (IQR 8–14) days, with a median intensive care unit stay of 2.0 (IQR 1.0–4.0) days.

## Safety and patient-reported outcomes

Postoperative safety outcomes are reported in Table 3. Thirty-day mortality was 1.8% (6). The cause of death was sepsis in a patient who underwent concomitant CABG with a complicated post-operative course, cardiogenic shock in a patient who underwent concomitant CABG, mitral and tricuspid valve treatment, subarachnoid hemorrhage in a patient requiring post-cardiotomy ECMO (Extra Corporeal Membrane Oxygenation), pneumonia and hemorrhagic shock in a patient who underwent concomitant CABG. In one patient, who underwent concomitant CABG, atrial fibrillation treatment and aortic root replacement, the reason of death was unknown. No early re-interventions were reported, neither case of valve thrombosis. Five strokes were reported (1.5%), among those one hemorrhagic. In 8 patients acute kidney failure (AKI) occurred (2.4%), only one **(0.3%)** requiring dialysis, and one myocardial infarction (0.3%). Thirteen cases of Atrio Ventricular (AV) block III were reported before 30 days (4.0%). A permanent pacemaker implant (PPI) was required in 13 (4.0%) patients. Distribution of pacemaker implant by valve size is reported in Table 3.

**Table 3:** Safety Outcomes

	**Early Incidence (≤ 30 days) (*N* = 328)**
All deaths	6 (1.8%)
Re-intervention	0 (0.0%)
Stroke	5 (15%)
TIA	0 (0.0%)
Acute Kidney Failure^a^	8 (2.4%)
Myocardial Infarction	1 (0.3%)
Endocarditis	0 (0.0%)
Bleeding	6 (1.8%)
AV block III	13 (4.0%)
Permanent Pacemaker Implant	13 (4.0%)
size S	3/65 (4.6%)
size M	5/98 (5.1%)
size L	2/98 (2.0%)
size XL	3/67 (4.5%)

*AV* Atrio-Ventricular

^a^Only one requiring renal replacement therapy

Out of the 328 patients enrolled in the study, 300 reached the 30-day follow-up visit at the time of this report.

**Fig. 2:**
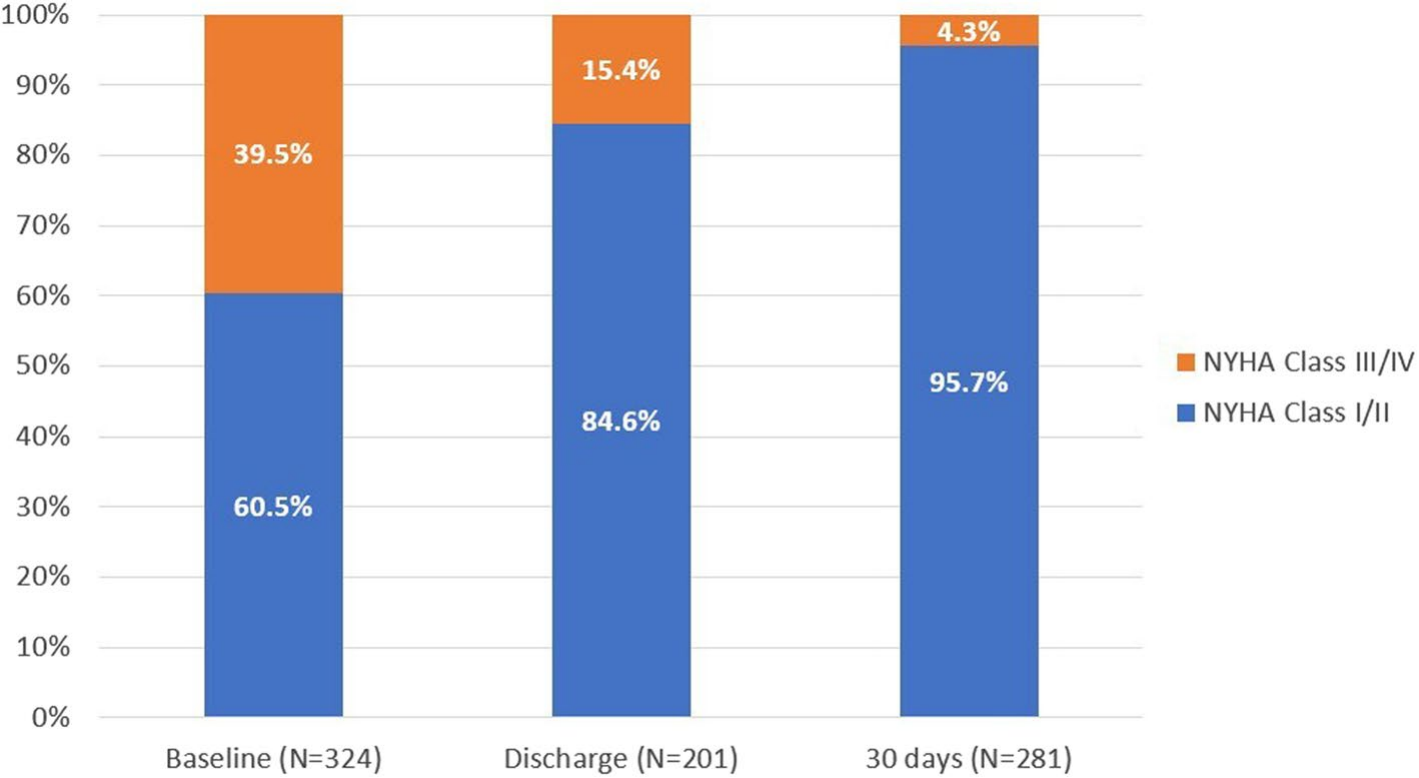
NYHA classification from preoperative to 30-days follow-up

The assessment of the functional status demonstrated marked and stable improvement of the NYHA class for most patients at the 30-day follow-up. Preoperatively, 196 patients (60.5%) were in NYHA functional class I or II versus 269 (95.7%) patients at the 30-day visit (Fig. 2).

**Fig. 3:**
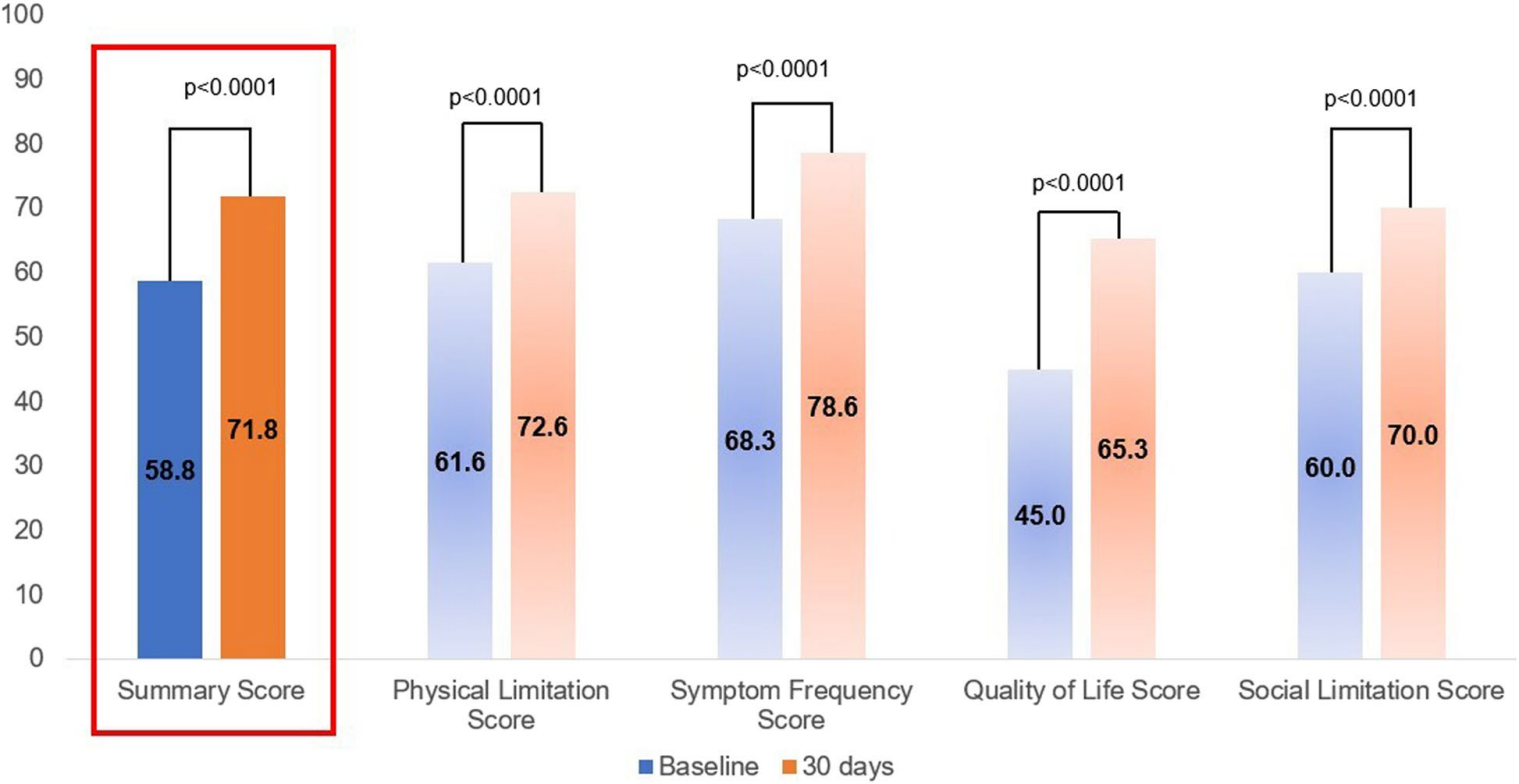
Mean KCCQ-12 scores at baseline and 30-days follow-up. The KCCQ-12 data were available for 326 patients (99.4%) at baseline and for 290 patients (96.7%) at 30-days

The KCCQ data were available for 326 patients (99.4%) at baseline and for 290 patients (96.7%) at 30-days. The KCCQ-12 summary score increased in the overall population from 58.8 ± 23.0 to 71.8 ± 22.1 at 30-days (*P* < 0.0001) (Fig. 3).

**Fig. 4:**
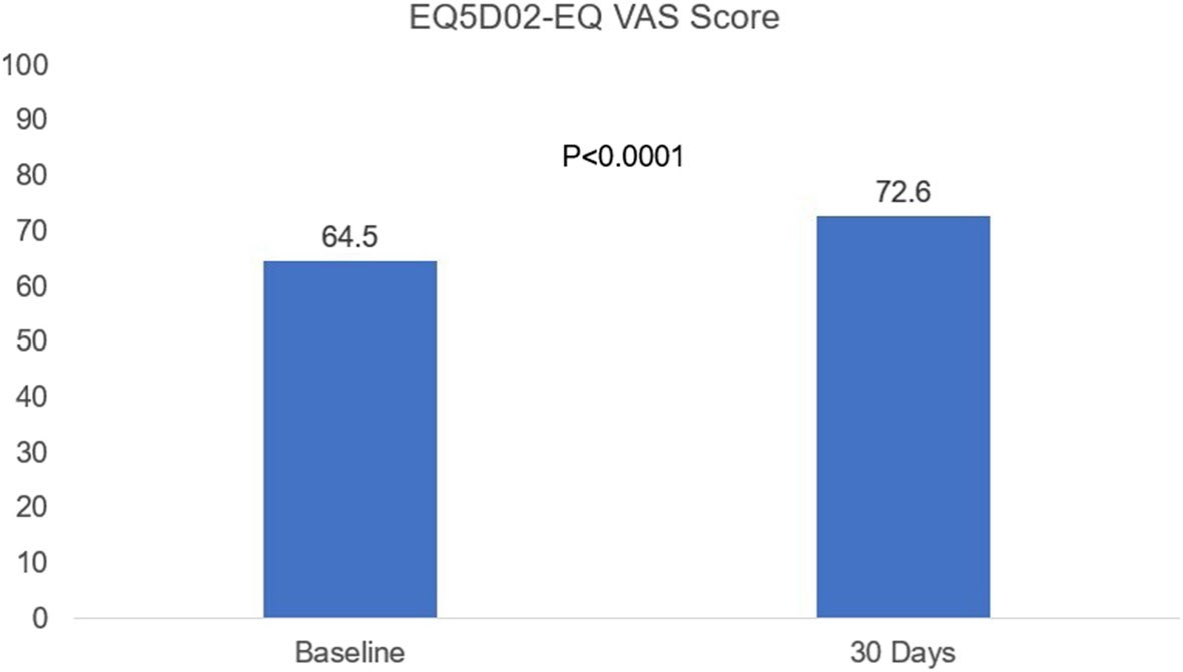
Mean EQ-5D-5L VAS Scores at baseline and 30-days follow-up. The EQ-5D-5L data were available for 323 patients (98.5%) at baseline and for 259 patients (86.3%) at 30-days. The score increased significantly from baseline to 30-days (*P* < 0.0001)

Quality of life was also measured using five-level EQ-5D (EQ 5D-5L) including both descriptive (index) and visual analogue scale (VAS) scores. The data were available for 323 patients (98.5%) at baseline and for 259 patients (86.3%) at 30-days. The baseline EQ-5D VAS score was 64.5 ± 20.4. The score increased significantly at 30-days (72.6 ± 17.5) (*P* < 0.0001) (Fig. 4 and Table S1).

### Echocardiographic results

Mean aortic pressure gradient decreased from 46.2 ± 17.3 mmHg preoperatively to 11.2 ± 5.0 mmHg at discharge and to 10.1 ± 4.7 mmHg at 30-days follow-up, and peak pressure gradient from 76.4 ± 28.0 mmHg preoperatively to 21.0 ± 9.1 and 18.7 ± 9.1 mmHg at discharge and 30 days, respectively.

The study demonstrated an increase in the mean valve effective orifice area (EOA) from 0.77 ± 0.23 cm^2^ before surgery to 1.73 ± 0.54 cm^2^ at discharge, which remained stable at 30-day follow-up. Severe patient prosthesis mismatch was found only in three patients implanted with Size S without any symptoms. Regression in left ventricular mass was observed, from 213.4 ± 64.7 g preoperatively to 188.6 ± 74.1 g already at the 30-day evaluation. The incidence of paravalvular leak greater than mild was very low at the 30-day follow-up, with only one case of moderate PVL (1/218, 0.5%) in a patient who underwent concomitant CABG in full sternotomy approach. No cases of moderate or severe central regurgitation were reported at 30-day (Table 4). Echocardiographic findings by valve size are reported in the supplemental material (Table S2).

**Table 4:** Site reported echocardiographic findings

	**Baseline**	**Discharge**	**30-days**
**Left Ventricular Ejection Fraction [%]**			
n	300	244	228
Mean ± SD	57.1 ± 9.8	56.6 ± 8.5	57.0 ± 7.3
Median	60.0	59.0	59.0
IQR	54.0;63.0	53.5;61.0	55.0;60.0
**Mean Pressure Gradient [mmHg]**			
n	296	257	224
Mean ± SD	46.2 ± 17.3	11.2 ± 5.0	10.1 ± 4.7
Median	44.0	11.0	9.8
IQR	37.0;56.3	8.0;14.0	7.0;12.6
**Peak Pressure Gradient [mmHg]**			
n	279	248	223
Mean ± SD	76.4 ± 28.0	21.0 ± 9.1	18.7 ± 9.1
Median	74.0	20.0	18.0
IQR	60.0;93.0	15.1;25.0	14.0;22.0
**Effective Orifice Area [cm2]**			
n	178	58	97
Mean ± SD	0.77 ± 0.23	1.73 ± 0.54	1.70 ± 0.54
Median	0.80	1.67	1.70
IQR	0.60;0.90	1.30;2.00	1.30;2.00
**Effective Orifice Area Index [cm2/m2]**			
n	76	44	74
Mean ± SD	0.46 ± 0.33	1.06 ± 0.36	0.98 ± 0.34
Median	0.40	1.00	0.91
IQR	0.34;0.48	0.82;1.19	0.80;1.12
**Left Ventricular mass [g]**			
n	99	64	88
Mean ± SD	213.4 ± 64.7	191.4 ± 66.3	188.6 ± 74.1
Median	204.5	187.8	184.2
IQR	168.1;239.9	140.7;230.1	137.7;221.6
**Paravalvular Regurgitation**		*N* available = 204	*N* available = 218
None	NA	193 (94.6%)	201 (92.2%)
Trace	NA	6 (2.9%)	9 (4.1%)
Mild	NA	5 (2.5%)	7 (3.2%)
Moderate	NA	0	1 (0.5%)
Severe	NA	0	0
**Central Regurgitation**	*N* available = 241	*N* available = 221	*N* available = 230
None	76 (31.5%)	197 (89.1%)	213 (92.6%)
Trace	33 (13.7%)	21 (9.5%)	13 (5.7%)
Mild	78 (32.4%)	3 (1.4%)	4 (1.7%)
Moderate	43 (17.8%)	0	0
Severe	11 (4.6%)	0	0

*SD* standard deviation, *IQR* interquartile range

## Discussion

This study reports some initial clinical and echocardiographic results with the Perceval PLUS sutureless valve from the MANTRA multicentric study. This study is a worldwide prospective observational registry on Corcym devices in multiple institutions where the devices are adopted in their daily clinical practice, to provide real-world evidence.

Aligned with previous published experience on the Perceval platform [11], these data demonstrate that the implantation of the new model, Perceval PLUS, is a safe and feasible procedure associated with low mortality, excellent hemodynamic performance, and significant improvement of the quality of life.

In this preliminary experience, early mortality was 1.8%, below than expected according to patient risk (mean EuroSCORE II 2.9 ± 3.9). None of the deaths were related to the device. At 30-days, low morbidity was observed; stroke was reported in 5 patients (1.5%), and AKI in 8 cases (2.4%). No re-interventions were reported. Median of intensive care unit stay was 2.0 days and total length of stay 10.0 days.

The KCCQ-12 summary score increased significantly at 30-days from baseline, showing a rapid improvement of the patient conditions, similar to what observed with Transcatheter Aortic Valve Replacement (TAVR) in low-risk patients, a recovery that was significantly faster than with traditional surgery [19]; this may suggest that shorter cross clamp and CPB time as well as MICS approach with sutureless technology can have a positive impact on faster patient recovery.

Reduction of mean transvalvular gradient as well as increase of effective orifice area was observed from baseline to discharge, and 30-day follow-up. Severe patient prosthesis mismatch, defined as effective orifice area index (EOAi) < 0.65 [20], was found only in three patients implanted with size S. These results compared with other series [11, 21, 22] are better, showing lower mean gradients at discharge (11.2 ± 5.0 mmHg) than the ones reported in the multicentric SURE-AVR study [11]. This is the first series including only subjects enrolled after the “sizing campaign” done by the Sponsor, that focused on avoiding oversizing and thus improving the hemodynamics and safety outcomes. These improvements in the clinical outcomes were also investigated by Szecel et al. [21], where they compared the Perceval results obtained before and after the change in the sizing procedure. The results showed a decreased pacemaker rate and improved hemodynamics, confirming the importance of avoiding oversizing, which is crucial for the best hemodynamic and clinical outcomes with the Perceval valve. This is also in line with the lowest incidence of permanent pacemaker implantation observed in this series compared to other published experiences [11, 23]. The reason behind this positive trend may be related to the improved design of Perceval PLUS, featuring a reduced sub annular valve collar protrusion in the size XL. While in the previous publications with Perceval the use of a valve size XL was an independent predictor of postoperative permanent pacemaker implant [23], in this preliminary experience the incidence of PPI with valve size XL was comparable to other valve sizes (4.5% size XL vs 4.6%, 5.1% and 2.0% with size S, M and L, respectively), suggesting the positive impact of the new design on this size.

Durability of the Perceval platform has been demonstrated by the 13-year experience published by Lamberigts et al. [24]. The Perceval Plus model features an innovative tissue treatment (FREE) that addresses both sources of tissue mineralization (phospholipids and aldehydes) [12], with the aim of increasing the durability of the bioprosthesis. No long term data are available today on the new Perceval PLUS, but we can observe a reduction of the mean age at the time of implant for patients enrolled in the MANTRA study (71.9 ± 6.4 vs 75.3 ± 7.0 in SURE-AVR) with more than 14% of the patients younger than 65 years, notoriously more prone to an accelerated calcification and higher incidence of prosthetic valve degeneration [25, 26]. The new anti-mineralization treatment of Perceval PLUS appeared to be safe and durable without any major signs of mineralization or degeneration in vitro and in animal studies [12].

The reduced time needed for the implantation is a potential advantage of this prosthesis. In this series, the overall cross-clamp time was 61.0 ± 28.8 min and pump time 92.9 ± 44.2 min, shorter than the values reported in the Society of Thoracic Surgeons database, where the cross-clamp and CPB time for AVR in full sternotomy are 77.9 and 106.4 min, respectively. In 72.7% of patients with isolated AVR, Perceval Plus was implanted via a minimally invasive approach, with even lower surgical timings (CPB time of 82.5 ± 30.5 min and cross-clamp of 52.8 ± 17.7 min). As the main drawbacks of minimally invasive AVR are the cardiopulmonary by-pass and cross-clamp times that are longer than in conventional surgery [2], we believe that sutureless technology, supporting intrinsically lower overall cross-clamp and pump times might be a viable solution in minimally invasive approaches; in the case of Perceval, these advantages were already described in the setting of mini-sternotomy [27] and right mini-thoracotomy [22]. The introduction of the new generation accessories (RelyON), featuring a low-profile valve delivery system, may further optimize visibility during minimally invasive approach.

### Study limitations

This study has the limitations of any observational study involving no adjudication of patient inclusion and adverse events. It is a prospective non-randomized study; therefore, lacking a comparative arm. Since follow-up visits were performed according to the site’s routine practice, not all the echocardiographic parameters were available for all the patients. The results are still preliminary since the enrollment is still ongoing.

## Conclusions

In conclusion, this multi-center, real-world experience with the Perceval PLUS valve showed early favorable hemodynamic results, good clinical outcomes, and moderate-to-large improvement of Quality of Life already at 30 days. Further clinical and echocardiographic evaluations with a larger patient cohort and a longer follow-up period will be necessary to confirm the findings of this preliminary experience.

### Supplementary Information

Supplementary Material 1.

## Data Availability

The datasets used and/or analysed during the current study are available from the corresponding author on reasonable request.

## Abbreviations

AVR: Aortic Valve Replacement
STS: Society of Thoracic Surgeons
NYHA: New York Heart Association
KCCQ: Kansas City Cardiomyopathy Questionnaire
CPB: Cardiopulmonary bypass
CABG: Coronary Artery Bypass Graft
AV: Atrio Ventricular
PPI: Permanent Pacemaker Implant
EOA: Effective Orifice Area
TAVR: Transcatheter Aortic Valve Replacement
