# Supplementary material for: Early outcomes of aortic valve replacement with Perceval PLUS sutureless valve: results of the prospective multicentric MANTRA study
Source: J Cardiothorac Surg. 2024 Jun 21;19:340. doi: 10.1186/s13019-024-02861-1 (PMC11191267; doi:10.1186/s13019-024-02861-1)
Supplement: Supplementary file 1 — Supplementary Material 1. [file 13019_2024_2861_MOESM1_ESM.docx]

**Perceval PLUS early outcomes in the MANTRA study**

**SUPPLEMENTARY APPENDIX**

**Contents**

[MANTRA Aortic sub-study INVESTIGATORS (implanting Perceval PLUS) AND CONTRIBUTORS 2](#_Toc143776015)

# MANTRA Aortic sub-study INVESTIGATORS (implanting Perceval PLUS) AND CONTRIBUTORS

**MANTRA STEERING COMMITTEE**

- Bart Meuris, UZ Gasthuisberg Leuven, University Hospital, Leuven, Belgium
- Patrizio Lancellotti, University Hospital Liège, CHU Sart Tilman, Liège,Belgium
- Serdar Günaydın, Ankara City Hospital, Ankara, Turkey
- Jörg Kempfert, Klinik für Herz-, Thorax- und Gefäßchirurgie Deutsches Herzzentrum der Charité, Berlin, Germany
- Gabriel S. Aldea, University of Washington, Seattle, WA, USA

**STUDY SITES, INVESTIGATORS AND KEY PERSONNEL**

| **Site** | **Personnel** |
| --- | --- |
| Dedinje Cardiovascular Institute, Milana Tepića 1, 11040 Belgrade Serbia | Principal Investigator  Slobodan Micovic  Sub-Investigators  Milan Milojevic, Tatjana Raickovic, Petar Vukovic |
| Hospital de Santa Maria Lisbon, Av. Prof. Egas Moniz MB, 1649-028 Lisbon, Portugal | Principal Investigator  Angelo Nobre  Sub-Investigators  Filipe Pereira, Ricardo Ferreira, Tiago Velho, Nadia Junqueira  Study Coordinators  Cecilia Gomes, Guilherme Da Silva |
| Seoul National University Hospital, 101, Daehak-ro Jongno-gu, 3080 Seoul, South Korea | Principal Investigator  Jae Woong Choi  Sub-Investigators  Yoonjin Kang  Study Coordinators  HaneSook Son |
| Ospedale del Cuore di Massa, Via Aurelia Sud, 54100 Massa, Italy | Principal Investigator  Marco Solinas  Sub-Investigators  Giovanni Concistrè, Sara Michelotti  Study Coordinators  Alessandra Parlanti |
| Department of Thoracic and Cardiovascular Surgery, West German Heart and Vascular Center Essen, University Hospital Essen.  Hufelandstraße 55,45147 Essen, Germany | Principal Investigator  Sharaf-Eldin Shehada  Sub-Investigators  Ali Haddad, Ilir Balaj  Study Coordinators  Marinela Jasarevic, Miralem Jasarevic, Sarah Jarkas, Maria Echterhoff |
| Department of Translational Medical Sciences, University of Campania “L. Vanvitelli” - Monaldi Hospital, Via Leonardo Bianchi, 80131 Napoli, Italy | Principal Investigator  Michele Torella  Sub-Investigators  Luigi Vitale  Study Coordinators  Maria Schioppo, Simona Schioppo |
| University Hospitals Cleveland Medical Center, 11100 Euclid Ave, Cleveland, OH 44106, USA | Principal Investigator  Cristian Baeza  Sub-Investigators  Marc Pelletier, Yasir Abu-Omar, Pablo Ruda Vega, Gregory Rushing, Lina El Hajj  Study Coordinators  Sarah Mitchell, Emily Mullenax, GaMia Dix, Mallory Lane Vaughn, Stacey Mazzurco, |
| UZ Leuven, University Hospital Leuven  Herestraat 49 3000, Leuven, Belgium | Principal Investigator  Bart Meuris  Study coordinators  Klaartje Van den Bossche, Joanna De Vis |
| Ochsner Clinic Foundation, 1514 Jefferson Highway, New Orleans, LA 70121, USA | Principal Investigator  Patrick E Parrino  Sub-Investigators  Aditya Bansal, Antonio Polanco, Stephen Spindel  Study Coordinators  Nicolle Scholl, Sylvia Laudun, Terri Lopez |
| Ospedale Ca’ Foncello di Treviso, Piazzale dell'Ospedale 1, 31100 Treviso, Italy | Principal Investigator  Giuseppe Minniti  Sub-Investigators  Antonio Pantaleo, Giulia Ballan |
| Klinikum Nürnberg, Paracelsus Medical University, Breslauer Str. 201, 90471, Nuremberg, Germany | Principal Investigator  Francesco Pollari  Sub-Investigators  Theodor Fischlein, Erik Bagaev, Islam Batashev, Christina Bermel, Matthias Fittkau, Faig Guliyev, Philipp Bauernschubert  Study coordinators  Fatima Jiven-Jetzelsberger, Apolonija Kalisnik |
| Fondazione Poliambulanza Istituto Ospedaliero, via Bissolati, 57, 25124 Brescia, Italy | Principal Investigator  Giovanni Troise  Sub-Investigators  Emmanuel Villa, Antonio Messina, Margherita Dalla Tomba |
| Herzzentrum Dresden GmbH Universitätsklinik  Fetscherstraße 76, 01307 Dresden, Germany | Principal Investigator  Utz Kappert  Sub-Investigators  Klaus Matschke  Study coordinators  Katrin Ploetze, Jeannette Schön, Christin Birkner, Denise Baumann |
| Klinikum Oldenburg GGMBH AoR, Rahel-Straus-Straße 10, 26133 Oldenburg, Germany | Principal Investigator  Friedrich Mellert  Sub-Investigators  Oliver Dewald, Mohammad Aldabbas  Study Coordinators  Bianca Sahlmann, Meike Ammersken |
| Pusan National University Yangsan Hospital, 20, Geum-ro, Mulgeum-eup, 50612 Yangsan, South Korea | Principal Investigator  Hyung Gon Je  Study Coordinators  Jeong Won Seo |
| Policlinico Paolo Giaccone, Via del Vespro 129, 90127 Palermo, Italy | Principal Investigator  Vincenzo Argano  Sub-Investigators  Sebastiano Castrovinci, Salvatore Torre, Enrico Amoncelli, Enza Tortorici, Salvatore Territo, Antonio Segreto |
| Catharina Ziekenhuis  Michelangelolaan 2, 5623 EJ Eindhoven, The Netherlands | Principal Investigator  Ka Yan Lam  Sub-Investigators  Erwin Tan, Mohammed Soliman  Study coordinators  Bianca De Louw, Marye Van Der Berg |
| A.O.U. Città della Salute e della Scienza di Torino - Ospedale Molinette, Corso Bramante, 88, 10126 Torino, Italy | Principal Investigator  Mauro Rinaldi  Sub-Investigators  Erik Cura Stura, Isabella Molinari |
| Ziekenhuis Oost Limburg, Schiepse Bos 6, 3600, Genk, Belgium | Principal Investigator  Herbert Gutermann  Study Coordinators  Karlien Seurs, Ann Steegmans |
| Policlinico S.Orsola-Malpighi, Via Giuseppe Massarenti 9, 40138, bologna, Italy | Principal Investigator  Davide Pacini  Sub-Investigators  Gianluca Folesani, Riccardo Nania, Elena Biagini |
| Az. Ospedaliero-Universitaria “Ospedali Riuniti” di Trieste, Via della Pietà 19, 34129 Trieste, Italy | Principal Investigator  Enzo Mazzaro  Sub-Investigators  Elisabetta Rauber, Ilaria Franzese, Angela Poletti |
| Inselspital, Universitätsspital Bern, Freiburgstrasse 18, CH-3010 Bern Switzerland | Principal Investigator  Matthias Siepe  Study Coordinators  Dorothee Keller |
| Quebec Heart and Lung Institute, Quebec, Canada  2725, chemin Sainte-Foy Québec (Québec), Canada | Principal Investigator  Pierre Voisine (former)  Maxime Laflamme  Sub-Investigators  Francois Dagenais, Dimitri Kalavrouziotis, Siamak Mohammadi, Eric Dumont  Study coordinators  Annie Bergeron, Nathalie Gagné, Francois Laforge, Joanie Lachance, Valerie Morin, Tea-Felicia Pilon |
| Duke University, 3580 White Zone, Duke South, 27710 Durham, USA | Principal Investigator  Chad Hughes  Sub-Investigators  Jeffrey Gaca, Carmelo Milano, Jacob Schroder, Donald Glower, John Haney, Adam Williams, Ryan Plitcha, Brittany Zwischenberger  Study coordinators  Sarah Casalinova. Shelly Fincannon, Susan Hajmohammad, Hillary Hood, Dana Giangiacomo, Mary Atkinson |
| Cleveland Clinic  2049 East 100th Street, Cleveland, OH 44195, USA | Principal Investigator  Eric Roselli  Sub-Investigators  Kevin Hodges, Faisal Bakaeen, Patrick Vargo, Haytham Elgharably, Marijan Koprivanac, Shinya Unai, Michael Zhen-Yu Tong  Study coordinators  Larissa Schaff, Mary Alice Bowman, Ravi Harley, Nicholas Fabrizio, Christina Stickan |
| St. Vincent Heart Center of Indiana  2001 West 86th Street, Indianapolis, Indiana 46260, USA | Principal Investigator  David Heimansohn  Sub-Investigators  Peter Walts, Sina Moanie, Giorgio Zanotti  Study coordinators  Jena Stanley, Hannah Willard, Deana Thomas, Kearsten McSwain, Jennifer Brown |
| King’s College Hospital, Denmark Hill, SE5 9RS London, UK | Principal Investigator  Max Baghai  Sub-Investigators  Alexandros Papachristidis  Study Coordinators  Sarah Byrne, Abigail Knighton, Jonathan Breeze, Mariya Tomi, Hosanna Assefa-Kebede |
| St. Michael's Hospital, 30 Bond St., Toronto, ON, Canada | Principal Investigator  Gianluigi Bisleri  Study Coordinators  Carlos Fernando |
| UZ Gent, De Pintelaan, 9000 Gent, Belgium | Principal Investigator  Thierry Bovè  Study Coordinators  Michael Buysse, Peter Vervaet |

**PROJECT MANAGEMENT (CORCYM S.r.l.)**

Elisa Cerutti, Sr Clinical Project Manager

Luca Foppoli, Manager, Clinical Operations

Silvia Dipinto, Clinical Project Manager

Nelly Rivera, Associate Project Manager

Michela Paroli, Associate Clinical Project Manager

Elona Mulai, Associate Clinical Project Manager

Mara Chiaro, Head Clinical, Quality and Regulatory Affairs

Sara Gaggianesi, Medical Affairs Director

Paola Morando, Principal Data Manager

Laura Chiara, Sr. Data Quality Specialist

**STATISTICAL and PROGRAMMING CONSULTANT**

Valos S.r.l, via Ceccardi 4/31, 16121, Genova, Italy

# Table S1: Health status (EQ-5D-5L) baseline and 30 days follow-up

|  | **Baseline** | **30 days** |
| --- | --- | --- |
| EQ-5D-5L Questionnaire Performed |  |  |
| YES | 323 | 259 |
| EQ5D02-Mobility |  |  |
| I have no problems in walking about | 127 (39.3%) | 131 (50.6%) |
| I have slight problems in walking about | 82 (25.4%) | 69 (26.6%) |
| I have moderate problems in walking about | 78 (24.1%) | 46 (17.8%) |
| I have severe problems in walking about | 34 (10.5%) | 13 (5.0%) |
| I am unable to walk about | 1 (0.3%) | 0 |
| Not Applicable | 0 | 0 |
| No Response | 1 (0.3%) | 0 |
| Multiple Responses Indicated | 0 | 0 |
|  |  |  |
| EQ5D02-Self-Care |  |  |
| I have no problems washing or dressing myself | 234 (72.4%) | 185 (71.4%) |
| I have slight problems washing or dressing myself | 42 (13.3%) | 52 (20.1%) |
| I have moderate problems washing or dressing myself | 31 (9.6%) | 18 (6.9%) |
| I have severe problems washing or dressing myself | 11 (3.4%) | 3 (1.2%) |
| I am unable to wash or dress myself | 2 (0.6%) | 1 (0.4%) |
| Not Applicable | 0 | 0 |
| No Response | 2 (0.6%) | 0 |
| Multiple Responses Indicated | 0 | 0 |
|  |  |  |
| EQ5D02-Usual Activities |  |  |
| I have no problems doing my usual activities | 128 (39.6%) | 113 (43.6%) |
| I have slight problems doing my usual activities | 70 (21.7%) | 82 (31.7%) |
| I have moderate problems doing my usual activities | 85 (26.3%) | 37 (14.3%) |
| I have severe problems doing my usual activities | 33 (10.2%) | 18 (6.9%) |
| I am unable to do my usual activities | 6 (1.9%) | 7 (2.7%) |
| Not Applicable | 0 | 0 |
| No Response | 1 (0.3%) | 1 (0.4%) |
| Multiple Responses Indicated | 0 | 1 (0.4%) |
|  |  |  |
| EQ5D02-Pain/Discomfort |  |  |
| I have no pain or discomfort | 150 (46.4%) | 120 (46.3%) |
| I have slight pain or discomfort | 92 (28.5%) | 93 (35.9%) |
| I have moderate pain or discomfort | 55 (17.0%) | 36 (13.9%) |
| I have severe pain or discomfort | 19 (5.9%) | 7 (2.7%) |
| I have extreme pain or discomfort | 5 (1.5%) | 2 (0.8%) |
| Not Applicable | 0 | 0 |
| No Response | 1 (0.3%) | 0 |
| Multiple Responses Indicated | 1 (0.3%) | 1 (0.4%) |
|  |  |  |
| EQ5D02-Anxiety/Depression |  |  |
| I am not anxious or depressed | 152 (47.1%) | 167 (64.5%) |
| I am slightly anxious or depressed | 87 (26.9%) | 54 (20.8%) |
| I am moderately anxious or depressed | 59 (18.3%) | 30 (11.6%) |
| I am severely anxious or depressed | 15 (4.6%) | 6 (2.3%) |
| I am extremely anxious or depressed | 8 (2.5%) | 2 (0.8%) |
| Not Applicable | 0 | 0 |
| No Response | 2 (0.6%) | 0 |
| Multiple Responses Indicated | 0 | 0 |
|  |  |  |
| EQ5D02-EQ VAS Score |  |  |
| n | 322 | 259 |
| Mean (SD) | 64.5 (20.4) | 72.6 (17.5) |
| Median | 70.0 | 79.0 |
| Q1; Q3 | 50.0; 80.0 | 60.0; 85.0 |
| Min; Max | 0; 100 | 0; 100 |

# Table S2 “Site reported echocardiographic data by valve size”

|  | **S** | **M** | **L** | **XL** |
| --- | --- | --- | --- | --- |
| **Discharge** | | | | |
| **Left ventricular ejection fraction (%)** | | | | |
| n | 45 | 72 | 73 | 54 |
| Mean±SD | 58.4 (7.3) | 58.6 (7.5) | 56.7 (8.7) | 52.3 (8.9) |
| Median | 60.0 | 60.0 | 59.0 | 55.0 |
| IQR | 55.0; 63.0 | 55.0; 62.5 | 51.0; 64.0 | 50.0; 60.0 |
| **Mean pressure gradient (mmHg)** | | | | |
| n | 50 | 72 | 75 | 59 |
| Mean±SD | 14.3 (5.4) | 11.8 (5.2) | 10.3 (4.1) | 8.9 (4.1) |
| Median | 14.0 | 11.0 | 10.0 | 8.8 |
| IQR | 11.0; 17.0 | 8.7; 14.5 | 7.0; 14.0 | 6.4; 11.0 |
| **Peak pressure gradient (mmHg)** | | | | |
| n | 46 | 69 | 76 | 56 |
| Mean±SD | 25.3 (7.6) | 22.5 (9.9) | 19.8 (7.9) | 17.6 (9.1) |
| Median | 25.0 | 21.0 | 19.5 | 16.3 |
| IQR | 19.4; 29.2 | 16.0; 26.0 | 14.7; 24.0 | 12.9; 21.3 |
| **Effective orifice area (cm^2^)** | | | | |
| n | 14 | 15 | 17 | 12 |
| Mean±SD | 1.5 (0.4) | 1.5 (0.3) | 1.9 (0.5) | 2.0 (0.8) |
| Median | 1.4 | 1.4 | 1.8 | 2.0 |
| IQR | 1.3; 1.8 | 1.3; 1.7 | 1.6; 2.2 | 1.4; 2.3 |
| **Effective orifice area index (cm^2^/m^2^)** | | | | |
| n | 12 | 11 | 13 | 8 |
| Mean±SD | 0.9 (0.3) | 0.9 (0.1) | 1.3 (0.4) | 1.1 (0.4) |
| Median | 0.9 | 0.8 | 1.2 | 1.0 |
| IQR | 0.8; 1.1 | 0.8; 1.0 | 1.1; 1.5 | 0.8; 1.2 |
| **30 days** | | | | |
| **Left ventricular ejection fraction (%)** | | | | |
| n | 42 | 66 | 66 | 54 |
| Mean±SD | 58.4 (7.1) | 58.6 (7.2) | 56.8 (6.2) | 54.5 (8.3) |
| Median | 59.0 | 60.0 | 58.5 | 55.0 |
| IQR | 55.0; 61.0 | 55.0; 60.0 | 55.0; 60.0 | 50.0; 60.0 |
| **Mean pressure gradient (mmHg)** | | | | |
| n | 41 | 66 | 64 | 53 |
| Mean±SD | 11.5 (4.1) | 11.1 (4.2) | 9.7 (5.9) | 8.375 (3.5) |
| Median | 11.0 | 10.4 | 9.0 | 8.1 |
| IQR | 9.0; 13.0 | 8.0; 14.0 | 6.4; 11.0 | 6.0; 10.0 |
| **Peak pressure gradient (mmHg)** | | | | |
| n | 41 | 67 | 66 | 49 |
| Mean±SD | 20.4 (7.0) | 20.2 (7.8) | 18.1 (12.1) | 16.1 (6.7) |
| Median | 19.360 | 20.000 | 16.500 | 15.0 |
| IQR | 16.000; 22.000 | 15.000; 24.800 | 14.440; 20.880 | 11.8; 20.0 |
| **Effective orifice area (cm^2^)** | | | | |
| n | 19 | 27 | 33 | 18 |
| Mean±SD | 1.4 (0.4) | 1.7 (0.4) | 1.8 (0.5) | 1.9 (0.7) |
| Median | 1.3 | 1.6 | 1.7 | 2.0 |
| IQR | 1.1; 1.7 | 1.3; 1.8 | 1.5; 2.1 | 1.7; 2.3 |
| **Effective orifice area index (cm^2^/m^2^)** | | | | |
| n | 15 | 20 | 23 | 16 |
| Mean±SD | 0.9 (0.3) | 0.9 (0.2) | 1.0 (0.5) | 1.0 (0.2) |
| Median | 0.9 | 0.9 | 1.0 | 1.0 |
| IQR | 0.7; 1.1 | 0.8; 1.1 | 0.8; 1.2 | 0.9; 1.2 |

SD: standard deviation, IQR: interquartile range
